# Yinzhihuang injection as adjuvant treatment for neonatal hyperbilirubinemia: a systematic review and meta-analysis of randomized clinical trials

**DOI:** 10.3389/fphar.2024.1467325

**Published:** 2024-12-05

**Authors:** Junqiang Li, Liyu Zhang, Zhongguo Chen, Min Qin

**Affiliations:** ^1^ Department of Neonatology, Lincang Maternity and Child Health Hospital, Lincang, Yunnan, China; ^2^ Administrative Department of Lincang Maternity and Child Health Hospital, Lincang, Yunnan, China

**Keywords:** neonatal hyperbilirubinemia, Yinzhihuang injection, systematic review, meta-analysis, paediatrics

## Abstract

**Background:**

Neonatal hyperbilirubinemia (NH) is a common phenomenon for neonate inpatients, and Yinzhihuang (YZH) injection is a well-known Chinese herbal formula for the treatment of NH. This study aims to evaluate the efficacy of YZH injection on NH.

**Methods:**

A comprehensive literature search was performed in four Chinese databases [China National Knowledge Infrastructure (CNKI), Chinese Biomedical Literature (CBM), China Science Journal Database (VIP), and Wan Fang ] and four English language databases (PubMed, Web of Science, EMbase and the Cochrane Library) from inception to 16 April 2024. Randomized controlled trials on YZH injection for NH were collected. Review Manager 5.4 software was used for meta-analysis and bias risk assessment.

**Result:**

Twenty-two trials involving 2,714 neonates were included. Significant differences were detected between YZH injection group and control group for total effective rate (RR = 1.15, 95%CI: 1.11 to 1.20, *p* < 0.00001), serum bilirubin level (the third day, MD = −36.80, 95%CI: −52.07 to −21.53; the fifth day, MD = −4.18, 95%CI: −7.50 to −0.86, *p* < 0.00001), and time to jaundice resolution (MD = −2.46, 95%CI -2.81 to −2.10, *p* < 0.00001). Adverse effects of YZH injection were described in four trials, with an incidence of 8.60%.

**Conclusion:**

The use of YZH injection as an adjuvant to NH appears to be safe and may offer superior benefits compared to routine treatment alone for NH. However, these potential benefits require confirmation in future trials employing rigorous methodology. The current evidence is not enough to recommend YZH injection as a routine treatment for NH.

**Systematic Review Registration:**

PROSPERO (https://www.crd.york.ac.uk/prospero/), identifier, CRD42024556811.

## Introduction

Neonatal hyperbilirubinemia (NH) is a common phenomenon, affecting about 60% of full-term and 80% of preterm neonates (Zhang et al., 2022). The condition arises from an imbalance in the production and elimination of unconjugated bilirubin (Cremer et al., 1958). Unconjugated bilirubin is a neurotoxin that can cause kernicterus and bilirubin encephalopathy, which may result in severe brain injuries and permanent neurodevelopmental damage. It is estimated that at least 481,000 full-term or near-full-term newborns worldwide suffer from severe hyperbilirubinemia each year. Of whom 114,000 died and more than 63,000 were moderately or severely disabled (Lawn et al., 2014; Bhutani et al., 2013).

According to American Academy of Pediatrics recommendations (Bhutani et al., 2013), both total bilirubin and transcutaneous bilirubin levels should be monitored during the evaluation of NH, and treatment should be considered for levels exceeding a specified threshold (Slusher et al., 2011). Phototherapy was first introduced 60 years ago (Cremer et al., 1958), and has since become the standard of care for NH, as it is noninvasive, acceptable, safe and effective (Hansen, 2010; Itoh et al., 2017). The indication for phototherapy is a rapidly rising or high serum total bilirubin level (American, 2004; Amos et al., 2017), with the aim of preventing neurotoxicity caused by unconjugated free bilirubin that crosses the blood-brain barrier. Nevertheless, it is important to be aware of the potential side effects associated with phototherapy. In addition to the potential for prolonged hospitalization, the short-term harms associated with phototherapy include erythematous rash, retinal damage, irritability, loose stools, dehydration, feeding difficulties, and the “bronze baby” syndrome (Maisels and Mcdonagh, 2008; Stokowski, 2011). A number of studies have indicated a potential association between phototherapy and an increased risk of infant and childhood cancer (Auger et al., 2019; Wickremasinghe et al., 2016), as well as an elevated prevalence of melanocyte nevi and a higher incidence of epileptic seizures during childhood (Maimburg et al., 2016; Newman et al., 2018).

YZH is a well-known traditional Chinese medicine (TCM) formula, composed of Herba Artemisiae Scopariae, Fructus Gardeniae, Radix Scutellariae and Flos Lonicierae Japonicae. It has been utilized in TCM for the treatment of jaundice for more than 1800 years (Wang, 2012). A pharmacological study has demonstrated that the principal components of this formula may inhibit hepatocyte apoptosis, promote the secretion and excretion of bile, and facilitate hepatic regeneration and the prevention of postoperative hepatic failure (Ogasawara et al., 2008). Previous studies conducted in China have demonstrated that YZH can effectively reduce the bilirubin level in hyperbilirubinemia and facilitate jaundice resolution (Chen et al., 2011; Chen, 2015). Deng and Li (2022) administered YZH orally to a mouse model of hemolytic jaundice and found that YZH had no significant effect on liver function, but had an improving effect on liver tissue damage. Some researchers compared the efficacy and safety of YZH combined with phototherapy and phototherapy in the treatment of NH (Dong, 2023; Ma et al., 2019) and found that the adverse reactions in the YZH combined with phototherapy group were significantly lower than those in the phototherapy group alone. The difference in adverse reactions between the two groups was not statistically significant, indicating that adding YZH granules to blue light irradiation therapy would not increase adverse reactions. It is speculated that the reason for this result may be that blue light irradiation will convert direct bilirubin into a substance called bilebrown, which takes a long time to achieve significant therapeutic effects in actual application. Blue light irradiation itself has a certain degree of irritation to children’s skin. After long-term use of blue light irradiation, children may have obvious fever, rash, and diarrhea symptoms. In addition, considering factors such as treatment time and irradiation wavelength, children may have severe complications such as hypocalcemia and bronze disease after blue light irradiation, which ultimately affect the treatment outcome of children. Wang et al. (2010) conducted a statistical analysis of 394 adverse reactions to YZH Injections reported in China between 1978 and 2008 and found that rash, diarrhea, and fever were the most common adverse reactions, which were relatively mild and improved after symptomatic treatment. However, the efficacy of intravenous YZH for NH remains uncertain. Therefore, this paper aimed to comprehensively assess the effectiveness of intravenous YZH injection on NH and to provide evidence-based recommendations for clinicians.

## Materials and methods

This study followed the Preferred Reporting Items for Systematic Reviews and Meta-Analyses flow diagram guidelines (Page et al., 2021). The protocol was registered on PROSPERO (CRD42024556811).

### Search strategy

Search of relevant studies were performed by two researchers. The databases included PubMed, Web of Science, EMbase, the Cochrane Library, CBM, Wanfang, VIP and CNKI databases up to 16 April 2024, with the search terms “Yinzhihuang injection”, “neonatal hyperbilirubinemia”, “neonatal jaundice”, and “randomized controlled trial”. Eligible studies were randomized controlled trials assessing the efficacy of YZH injection on NH in comparison with other treatments. Detailed search strategies is shown in Supplementary Appendix.

### Inclusion criteria

Prospective randomized controlled trials were eligible for inclusion. The study included infants within 28 days of birth who exhibited hyperbilirubinemia, defined as a direct bilirubin level exceeding 17.1 μmol/L, a total serum bilirubin level exceeding 85 μmol/L and a direct bilirubin level exceeding 20% of the total serum bilirubin or clinical features of visible jaundice in the sclera, body or limbs or other organs. Hyperbilirubinemia was considered regardless of the underlying cause, including physiologic or pathologic factors. The diagnostic criteria for NH followed international guidelines (Amos et al., 2017; American, 2004). YZH injection was in combination with routine therapy as a treatment group, compared with the routine therapy as a control group. The course of treatment with YZH is 3∼10 days. Routine therapies included phototherapy, phenobarbitone, albumin injection, or immune globulin injection. Outcome assessment should include at least one of the following indicators: overall effectiveness, serum bilirubin level and time to resolution of jaundice.

### Exclusion criteria

We excluded cohort studies, case control studies, case report/series, and review studies. Studies in which the outcome measurements could not be synthetically evaluated were also excluded.

### Data extraction

Data were summarized in a pre-designed form by two authors, and any difference was resolved by discussion. The following information was extracted: first author, publication date, sample size, sex, birth weight, gestational age, serum bilirubin at inclusion, interventions in the treatment and control groups and outcomes.

### Quality assessment

Two authors used the Cochrane Collaboration’s “Risk of Bias” tool (Higgins et al., 2008) to conduct the quality assessment. Seven items including random sequence generation, allocation concealment, blinding of participants and personnel, blinding of outcome assessment, incomplete outcome data, selective reporting and other bias were assessed as “low risk”, “high risk”,or “unclear risk”. Any disagreement was resolved by discussion with the third author.

### Statistical analysis

The Review Manager 5.4 (Cochrane Library Software, Oxford, United Kingdom) was used for meta-analysis. Risk ratio (RR) was calculated for dichotomous outcome. Mean difference (MD) were calculated for continuous data. The heterogeneity was tested by the χ^2^ test and the I^2^ statistic. If a significant heterogeneity existed (I^2^ ≥ 50% or *p* < 0.10), a random effect model was employed. Otherwise, the fixed effect model was selected (Higgins and Thompson, 2002). When significant heterogeneity is present, we performed sensitivity analysis and subgroup analysis to identify potential sources of heterogeneity. Funnel plots (Macaskill et al., 2001; Egger et al., 1997) were applied to detect publication bias if there were more than ten trials in a meta-analysis.

## Results

### Characteristics of included studies

**FIGURE 1:**
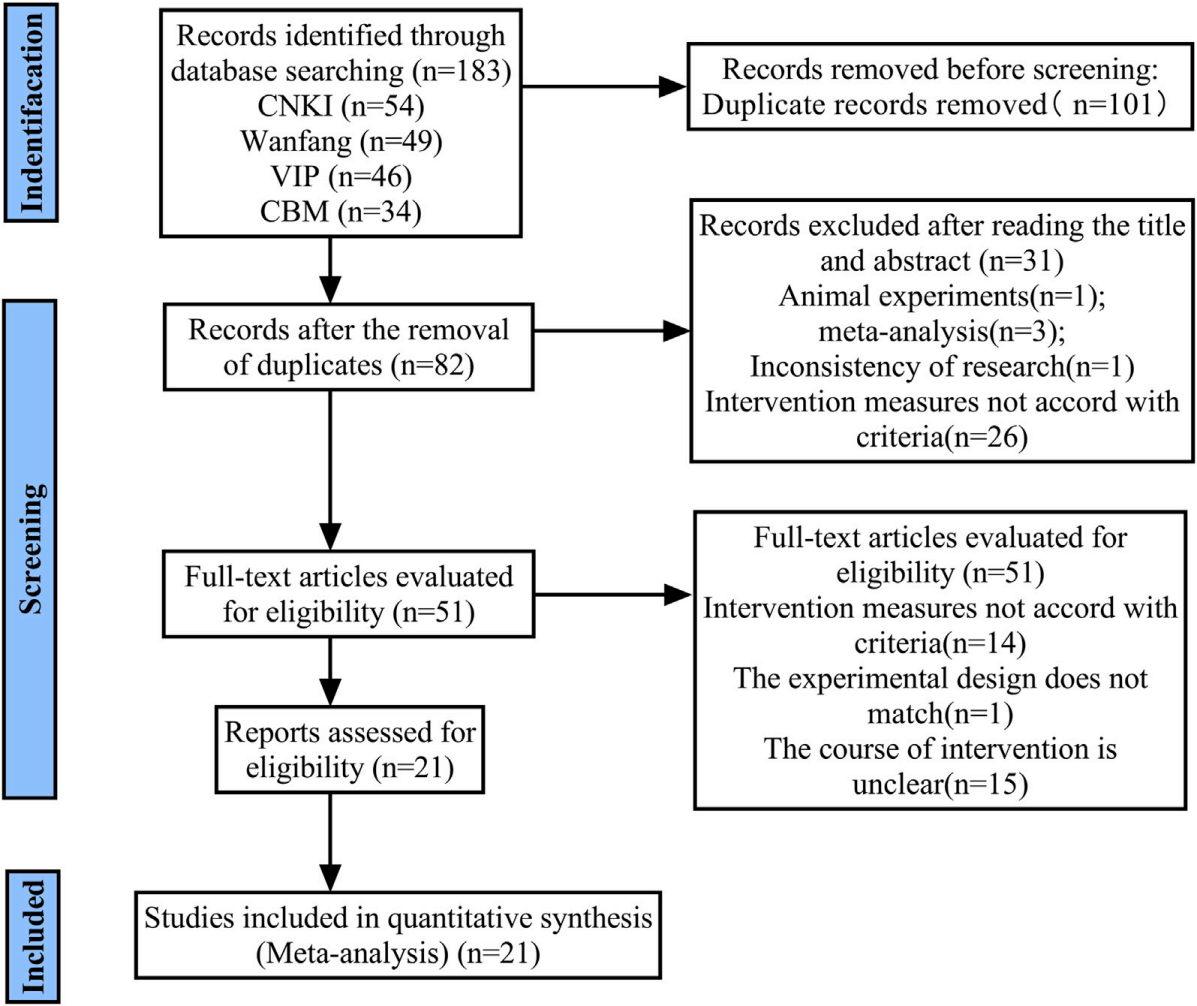
Flow chart of study selection.

A preliminary search yielded 183 citations. A total of 101 articles remained following the removal of duplicates. Following the initial screening of the titles and abstracts, 31 articles were excluded. A further 51 full-text articles were then assessed in more detail, with a further 30 articles being excluded. Finally, 21 studies met the inclusion criteria and were included in the systematic review (Figure 1).

These 21 included trials, which involved 2,714 NH participants (1,335 in the treatment group and 1,379 in the control group), were all conducted in China. They were published from 2004 to 2024, with sample size ranging from 36 to 400. Participants in the treatment group received YZH injection in combination with routine therapy, and were compared with routine therapy in controls. The characteristics of included studies are summarized in Table 1.

**TABLE 1:** Characteristics of included trials.

Author	Gestational age (weeks)		Birth weight (kg)		Age at inclusion (days)		Male/female		Serum bilirubin at inclusion (umol/L)		Intervention		Dose and course of yinzhihu-ang injection	Outcome measures
	T	C	T	C	T	C	T	C	T	C	T	C		
Cao (2015)	—	—	—	—	11.4 ± 1.9	10.7 ± 2.2	118/82	104/96	—	—	RT + IDYI	RT	5–10 mL, once a day, 3–7 days	①
Ding (2016)	—	—	—	—	—	—	32/28	33/27	—	—	Phototherapy + IDYI	Photothe-rapy	1 mL/kg, once a day, 6 d	①
Dou and Zhu (2015)	39.45 ± 4.29	39.28 ± 3.91	3.000 ± 0.340	3.029 ± 0.300	—	—	25/20	24/21	231.34 ± 14.98	233.89 ± 13.98	RT + IDYI	RT	5 mL, once a day, 7 d	①③
Fang et al. (2005)	37–42	37–41	2.85–4.2	2.8–4.05	2–28days, average 8.2 d	2–27days, average 7.9 d	22/20	20/18	283.6 ± 58.2	285.3 ± 55.2	RT + IDYI	RT	5 mL, once a day, 5∼7 d	②③
Gu et al. (2011)	37.50 ± 1.21	37.54 ± 1.25	3.01 ± 0.56	2.98 ± 0.60	4.50 ± 2.10	4.50 ± 2.20	45	48	293.90 ± 45.21	290.90 ± 47.38	RT + IDYI	RT	10 mL, 7–10 d	①
Huang et al. (2007)	38.5 ± 3.5	37.5 ± 4.2	2.900 ± 0.3	3.0 ± 0.26	1–28days, average 7.1 d	1–28days, average 7.1 d	19/16	18/17	299.70 ± 55.10	289.28 ± 58.30	RT + IDYI	RT	1∼2 mL/kg, once a day, 5 d	②
Ke et al. (2003)	37.59 ± 3.40	37.88 ± 2.92	2.9 ± 0.66	2.9 ± 0.68	3.18 ± 1.93	3.18 ± 1.96	38/32	36/30	260.38 ± 48.23	258.66 ± 45.63	RT + IDYI	RT	1 mL/kg, once a day, 3∼5 d	①③④
Li and Chen (2019)	—	—	—	—	—	—	28/27	30/25	257.25 ± 44.35	258.28 ± 48.56	Phototherapy + IDYI	Photothe-rapy	5–10 mL, 7 d	①②③④
Li and Cai (2012)	—	—	—	—	5.36 ± 1.87	5.42 ± 2.03	61/59	58/62	278.46 ± 41.47	279.12 ± 46.58	RT + IDYI	RT	1∼2 mL/kg, once a day, 5 d	①②③
Li (2014)	—	—	3.46 ± 0.41	3.52 ± 0.39	4.53 ± 2.56	4.53 ± 2.69	38/12	35/15	292.1 ± 3.2	297.6 ± 4.7	RT + IDYI	RT	1∼2 mL/kg, once a day, 5 d	①②③
Li (2006)	37.23 ± 4.55	37.44 ± 6.75	3.78 ± 2.79	3.28 ± 4.63	—	—	18	18	252.80 ± 25.44	254.64 ± 27.96	RT + IDYI	RT	10 mL, once a day, 10 d	③
Li (2016)	—	—	—	—	5.44 ± 1.86	5.47 ± 1.84	42/38	43/37	—	—	RT + IDYI	RT	1∼2 mL/kg, once a day, 5 d	①②③④
Li (2014)	—	—	—	—	—	—	48	48	292.10 ± 36.12	287.12 ± 35.95	Phototherapy + IDYI	Photothe-rapy	1∼2 mL/kg, once a day, 3 d	①②③
Liu (2016)	—	—	—	—	2.5 ± 1.1	2.8 ± 1.2	25/20	28/17	278.5 ± 2.1	279.1 ± 1.2	RT + IDYI	RT	1 mL/kg, 5 d	①②③
Wan (2007)	37.8 ± 3.8	38.5 ± 3.7	2.96 ± 0.263	3.020 ± 0.235	16 h–24days, average 5.4 d	15 h–22days, average 5.2 d	32/13	27/15	290.31 ± 61.52	286.32 ± 63.51	RT + IDYI	RT	4 mL, once a day, 5 d	②
Wang (2009)	37.18 ± 3.04	37.56 ± 3.22	3.01 ± 0.56	2.95 ± 0.60	3.18 ± 1.89	3.20 ± 1.90	24/18	21/17	265.21 ± 70.65	264.18 ± 58.12	RT + IDYI	RT	5∼6 mL (2 mL/kg), once a day, 7–10 d	①②③④
Wei (2009)	—	—	—	—	—	—	59/41	52/48	—	—	RT + IDYI	RT	5–10 mL, once a day, 3–7 days	①
Wu and Xiang (2017)	—	—	—	—	—	—	50/39	42/47	257.18 ± 44.38	258.27 ± 48.57	Phototherapy + IDYI	Photothe-rapy	5–10 mL, 7 d	①②③④
Xu and Lin (2009)	39.36 ± 4.2	38.80 ± 3.90	3.154 ± 0.31	3.178 ± 0.315	2–27days, average 6.2 d	2–27days, average 5.9 d	26/24	23/27	—	—	RT + IDYI	RT	1∼2 mL/kg, once a day, 3∼5 d	①
Zhan and Zhang (2011)	—	—	—	—	—	—	110	110	289.37 ± 37.43	288.32 ± 36.54	RT + IDYI	RT	1∼2 mL/kg, once a day, 5 d	①②③
Zhang and Feng (2008)	—	—	—	—	—	—	20/16	20/L7	298.56 ± 61.17	293.45 ± 59.36	RT + IDYI	RT	2 mL/kg, once a day, 5∼7 d	②

Note: T: Treatment group, C:Control group, RT: routine treatment, IDYI: intravenous drip of Yinzhihuang injection, /: not reported.

①:Efficacy, ②: time to jaundice resolution, ③: serum bilirubin level after treatment, ④:adverse effects.

### The quality assessment

We rated random sequence generation as low risk of bias if the method for random sequence generation was described as a random number table, computer-generated, coin tossing, shuffling cards or envelopes, throwing dice, drawing of lots or minimization. We rated random sequence generation as high risk of bias if the method of generation was not random. We rated random sequence generation as unclear risk of bias if insufficient information was reported to allow for a judgement. Allocation concealment was judged as adequate if allocation concealment was clearly described and an appropriate way of concealment was used, e.g., sequentially-numbered, opaque, sealed envelopes and central randomisation.

**FIGURE 2:**
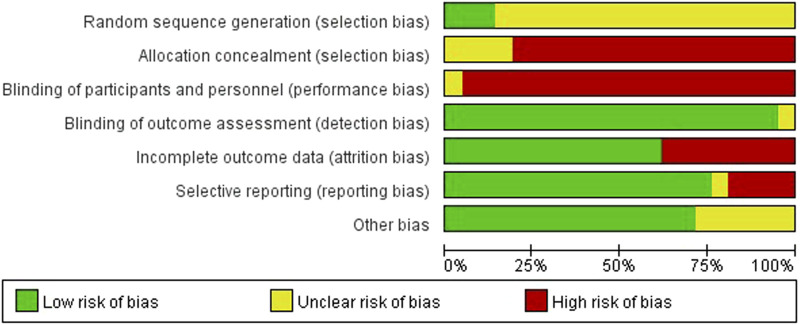
Risk of bias summarized.

**FIGURE 3:**
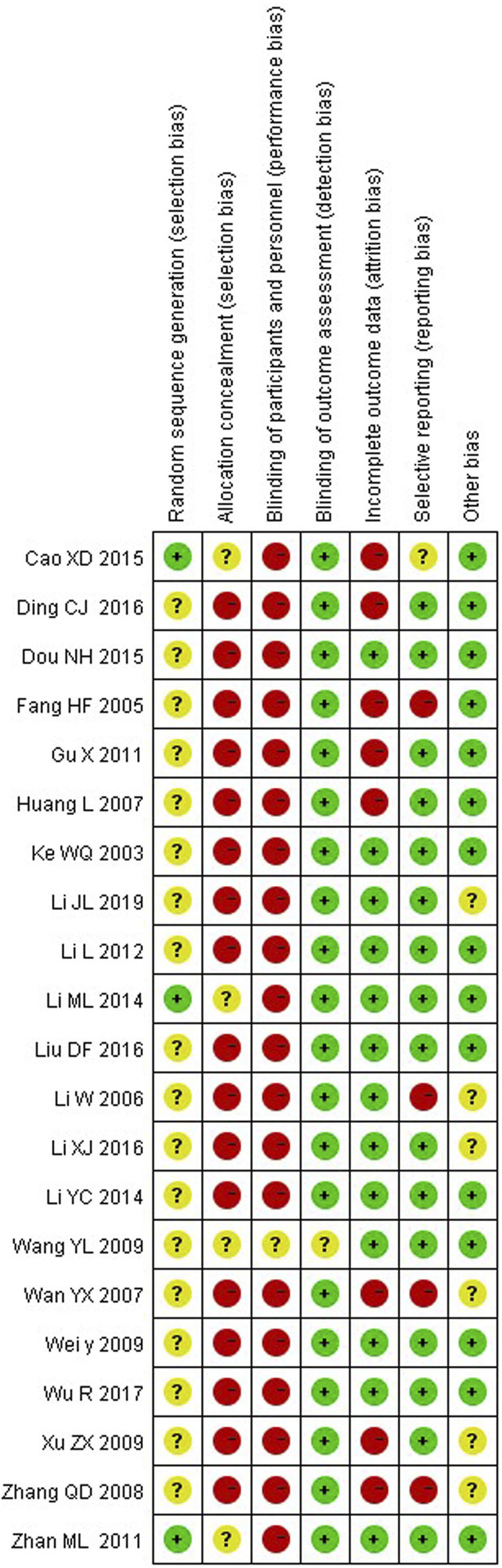
Methodological quality assessment of the risk of bias for each included study.

The quality assessment twenty out of twenty-one studies mentioned random sequence generation, of which three studies (Cao, 2015; Li, 2014; Zhan and Zhang, 2011) mentioned the random number table method, which had a high risk of bias in random sequence generation (selection bias). Four studies (Cao, 2015; Li, 2014; Zhan and Zhang, 2011; Wang, 2009) assigned hidden risks that were unclear, and the remaining studies were high risk. No trial blinded participants or personnel. Eight studies (Cao, 2015; Fang et al., 2005; Ding, 2016; Gu et al., 2011; Huang et al., 2007; Xu and Lin, 2009; Zhang and Feng, 2008; Wan, 2007) had incomplete outcome data and four studies (Fang et al., 2005; Wan, 2007; Zhang and Feng, 2008; Li, 2006) had selective reporting. Overall, the methodological quality of included studies was low (Figures 2, 3).

### Efficacy of YZH injection on NH

Therapeutic efficacy criteria (Han, 2012): (1) significant efficacy: the yellowing of the skin and mucous membranes subsided after treatment, and the serum total bilirubin level was reduced to normal; (2) efficacy: the yellowing of the skin and mucous membranes subsided partially, and the serum total bilirubin level was still higher than the normal level; (3) inefficacy: the yellowing of the skin and mucous membranes did not subside significantly, and the serum total bilirubin did not decrease significantly. Total effective rate = (markedly effective + effective) cases/total cases × 100%.

**FIGURE 4:**
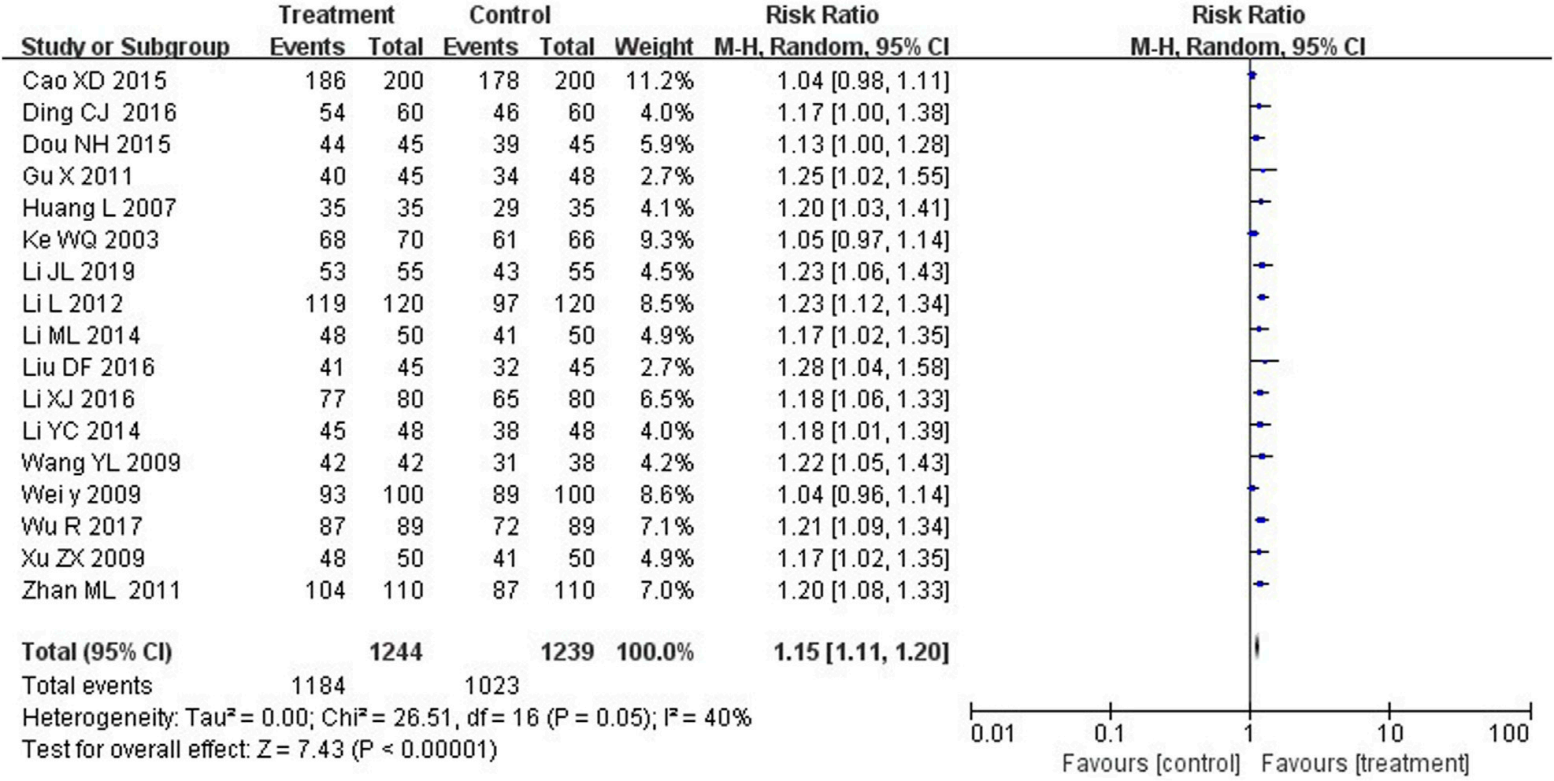
Forest plot of total effective rate.

Seventeen studies compared the total efficacy rate between the two groups. The results suggested that treatment group with YZH injection reported more effective cases than control groups, with a statistically significant difference (RR = 1.15, 95%CI: 1.11 to 1.20, *p* < 0.00001) (Figure 4).

### Serum total bilirubin levels

Serum total bilirubin levels were measured through automated biochemical profiling, and the unit of bilirubin concentration was micromoles per liter.

**FIGURE 5:**
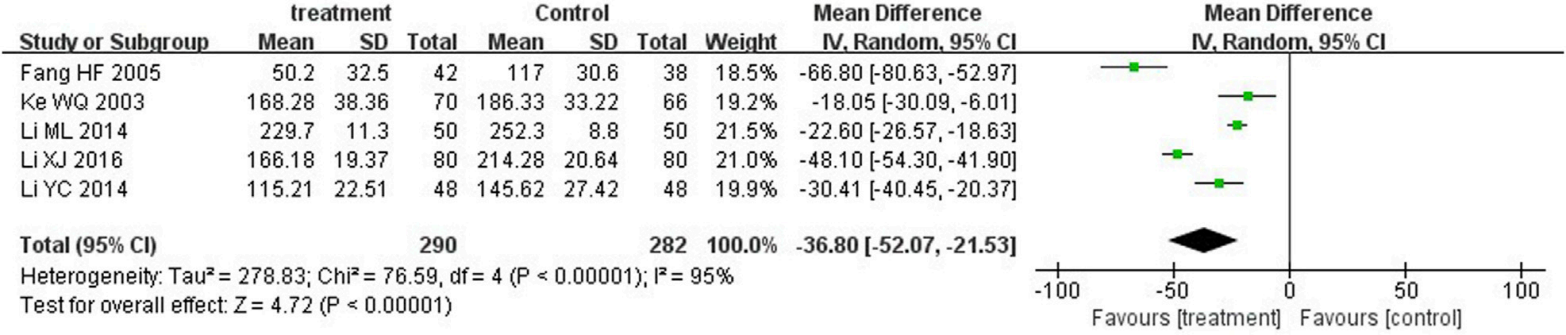
Forest plot of bilirubin levels at the third day.

**FIGURE 6:**
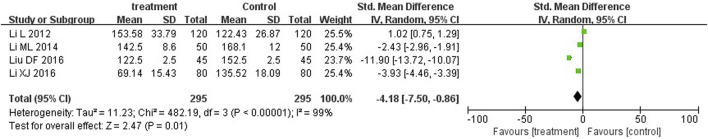
Forest plot of bilirubin levels at the fifth day.

Five trials (Fang et al., 2005; Ke et al., 2003; Li, 2014; Li, 2016; Li, 2014) and four trials (Li and Cai, 2012; Li, 2014; Li, 2016; Liu, 2016) reported serum total bilirubin levels after three-day and five-day treatment between two groups. A statistically significant reduction in bilirubin levels was observed at the third day in the treatment group in comparison with the control group (MD = −36.80, 95%CI: −52.07 to −21.53, *p* < 0.00001). On the fifth day, there was also significant differences between the two groups (MD = −4.18, 95%CI: −7.50 to −0.86, *p* = 0.01) (Figures 5, 6).

### Time to jaundice resolution

**FIGURE 7:**
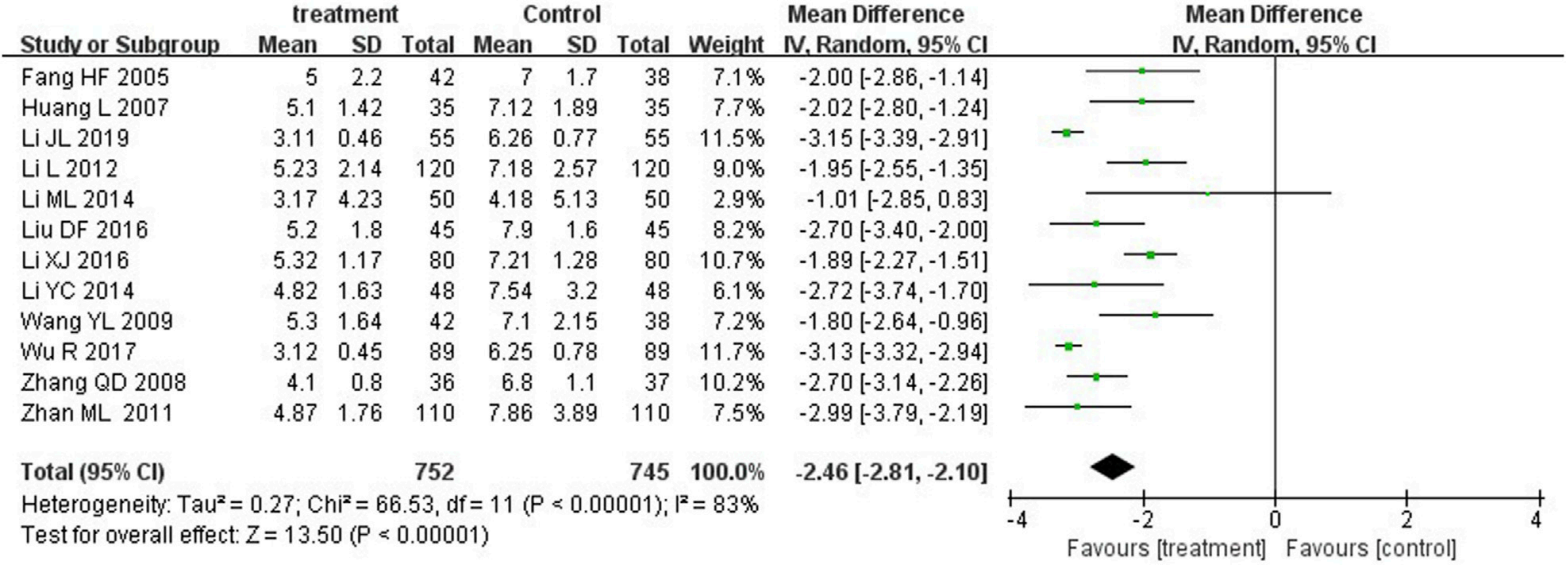
Forest of time to jaundice resolution.

Jaundice resolution, defined as the significant resolution of yellow coloration in the skin and/or sclera after treatment, or serum total bilirubin <2.5 mg/dL (Ruo-Han et al., 2018; Eaton et al., 2021). Twelve studies reported time to jaundice resolution. The MD was −2.46 (*p* < 0.00001) with a 95%CI of −2.81 to −2.10. Therefore, YZH injection group and control group showed significant differences in their ability to reduce the serum bilirubin concentration in patients (Figure 7).

### Adverse reactions

Four trials (Wu and Xiang, 2017; Li and Chen, 2019; Wang, 2009) reported adverse reactions after YZH injection. In total, 17 patients experienced adverse reactions (8.60% of the 186 patients treated). Of these, there were 8 cases of diarrhoea, 2 cases of fever, 4 cases of rash and 2 cases of irritability. The adverse reactions were not serious and improved with the administration of symptomatic treatment.

### Subgroup analysis

**FIGURE 8:**
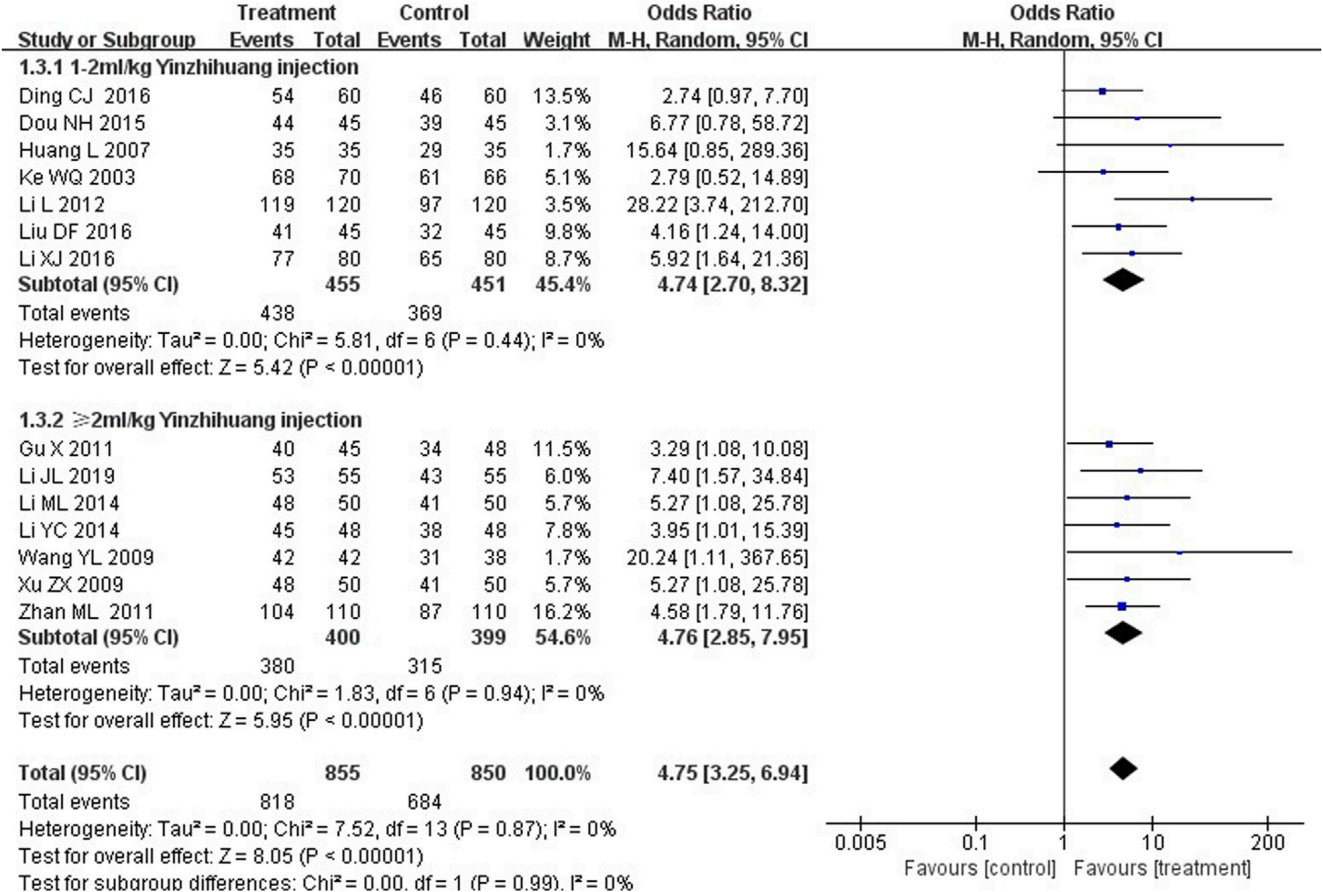
Subgroup analysis of total effective rate based on the dosage of YZH injection.

**FIGURE 9:**
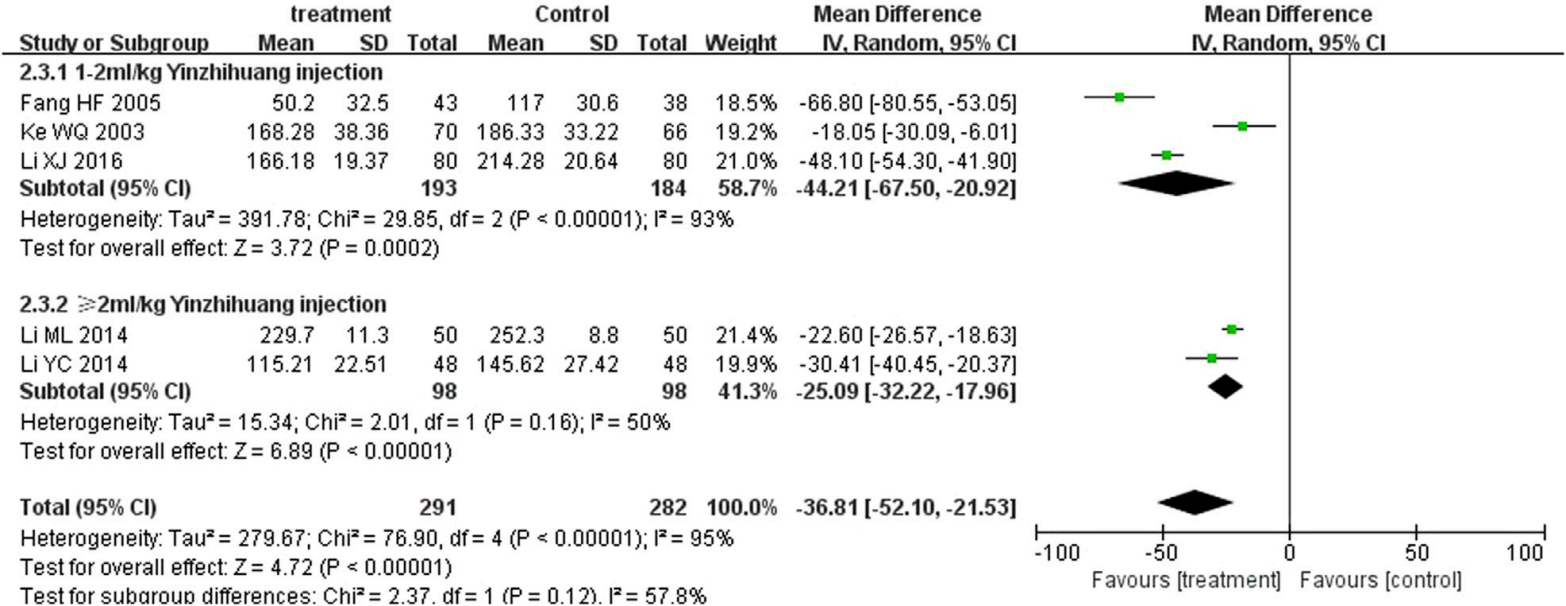
Subgroup analysis of total serum bilirubin levels at the third day based on the dosage of YZH injection.

**FIGURE 10:**
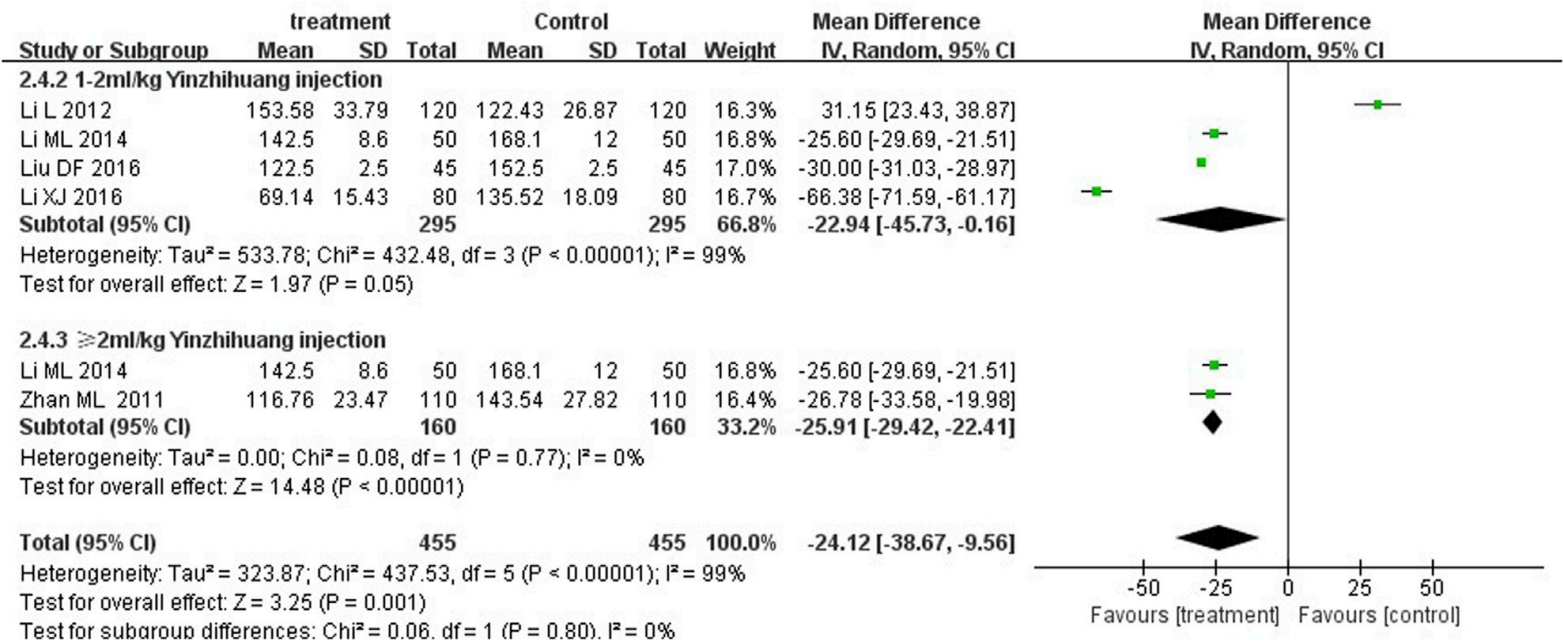
Subgroup analysis of total serum bilirubin levels at the fifth day based on the dosage of YZH injection.

**FIGURE 11:**
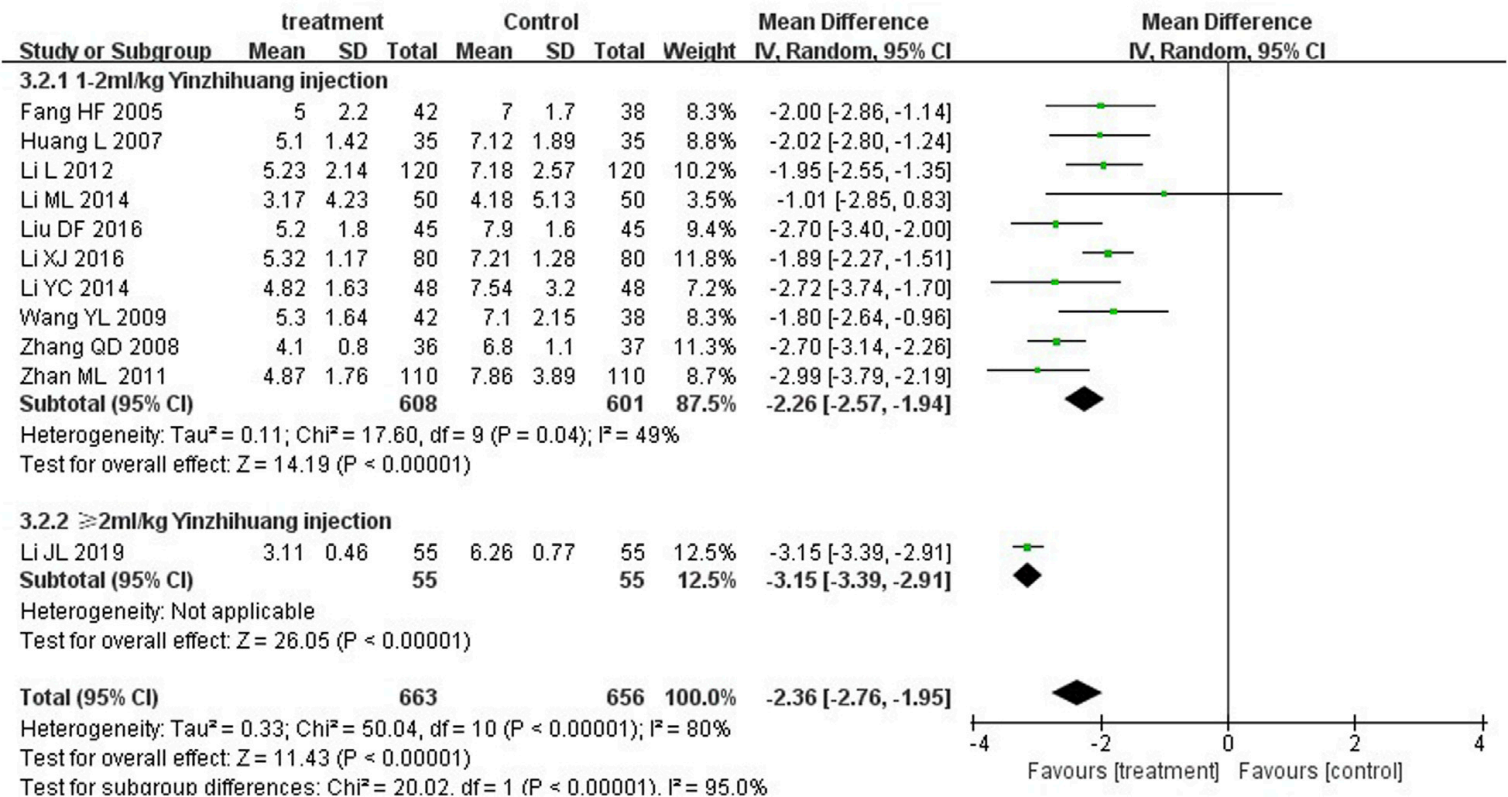
Subgroup analysis of time to jaundice resolution at the fifth day based on the dosage of YZH injection.

We performed the subgroup analysis based on the dosage of YZH injection (1–2 mL/kg and ≥2 mL/kg) to compare the efficacy, the total bilirubin level after treatment and jaundice resolution time between two groups. The subgroup analysis results also revealed that combination therapy was more efficacious than routine treatment. For those receiving 1–2 mL/kg, the MD was 4.74 (95% CI: 2.70 to 8.32, I^2^ = 0%), while for those receiving ≥2 mL/kg, the MD was 4.76 (95% CI: 2.85 to 7.95, I^2^ = 0%) (Figure 8). Both YZH injection of 1–2 mL/kg (third day, MD = −44.21, 95%CI: −67.50 to −20.92, I^2^ = 93%; fifth day, MD = −44.21, 95%CI: −67.50 to −20.92, I^2^ = 99%) and of ≥2 mL/kg (third day, MD = −25.09, 95%CI: −32.22 to −17.96, I^2^ = 50%; fifth day, MD = −25.09, 95%CI: −32.22 to −17.96, I^2^ = 50%) resulted in a significantly reduced serum total bilirubin levels (Figures 9, 10). The pooled data also suggested that YZH injection effectively decreased the time to jaundice resolution at 1–2 mL/kg (MD = −2.26, 95%CI: −2.57 to −1.94, I^2^ = 49%) and ≥2 mL/kg (not applicable) (Figure 11).

### Publication bias

**FIGURE 12:**
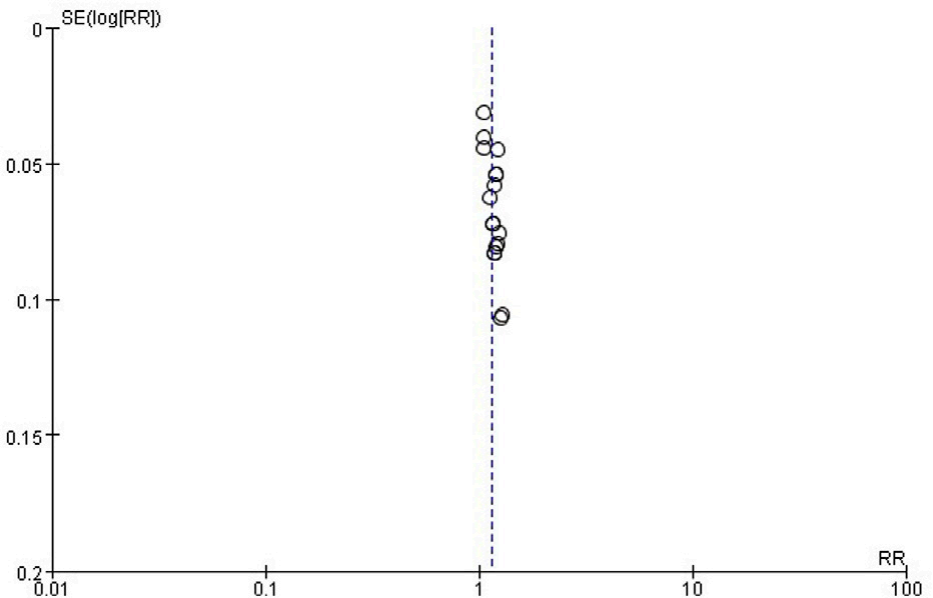
Funnel plot of Effective rates.

**FIGURE 13:**
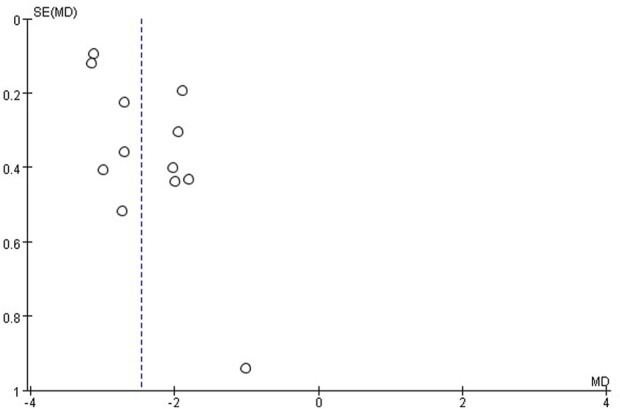
Funnel plot of time to jaundice resolution.

The publication bias of these studies was assessed by funnel plots. Asymmetric funnel plots reporting efficiency and time to resolution of jaundice indicated possible publication bias (Figures 12, 13).

## Discussion

The objective of this study is to evaluate the efficacy and safety of YZH injection as an adjuvant treatment for NH. The meta-analysis included 21 RCTs with a total of 2,714 participants. A total of 1,335 patients were treated with YZH injection, while 1,379 patients served as the control group. The majority of the included RCTs were deemed to have a high risk of bias. Seventeen and twelve studies, respectively, reported efficacy and time to jaundice resolution. A total of five and four studies, respectively, reported serum total bilirubin levels on days three and five of the treatment period. Three studies documented the occurrence of side effects. The results demonstrated that the treatment group exhibited a higher efficacy rate of therapy in comparison to the control group. Furthermore, the treatment group demonstrated a significantly reduced bilirubin level in comparison to the control group throughout the course of the treatment period. Furthermore, the treatment group exhibited a notable reduction in the duration of jaundice, accompanied by a low incidence of mild adverse effects. The administration of YZH injection may be considered as an adjuvant therapy in the treatment of NH, but the current evidence is not enough to recommend YZH injection as a routine treatment for NH. Given that it is a safer, more cost-effective, and more readily applicable option in clinical settings.

A systematic review of TCM for the treatment of neonatal jaundice indicated that a combination of YZH and routine treatment (phenobarbital, probiotics or phototherapy) may be more beneficial than routine treatment alone for the treatment of neonatal jaundice (Zhu et al., 2013). YZH is available in both oral and intravenous forms. Although there have been numerous studies on the use of oral YZH granules or injections in the treatment of NH, there is a paucity of research on the intravenous administration of YZH injection for the management of NH. The objective of our study is to address this gap in the existing literature. Our findings suggest that YZH injection has a synergistic effect on routine treatment in reducing serum bilirubin, attaining jaundice resolution and shortening the time to jaundice resolution. These results are consistent with those of previous studies.

The theory of TCM posits that neonatal jaundice is associated with innate factors, which are believed to be present at birth. The main syndrome in TCM that is used to describe different conditions of the same disease is fetal jaundice with the syndrome of stagnation and steaming of damp-heat, internal retention of cold wetness, or syndrome of qi stagnation and blood stasis. YZH is primarily composed of four principal components. The formula comprises the following ingredients: Artemisiae Scopariae Herba, Scutellariae Radix, Lonicerae Japonicae Flos, and Gardeniae Fructus. Artemisiae Scopariae Herba is an ancient TCM that is primarily utilized to address “damp-heat style” jaundice, hepatitis, heatstroke, and allergic inflammatory dermatitis. Randomized controlled clinical trials have demonstrated that this drug preparation can significantly reduce the recovery time of serum total bilirubin and the regression time of jaundice in the treatment of neonatal jaundice (Cai et al., 2020).

Scutellariae Radix is the root of Scutellaria baicalensis Georgi, which belongs to the Lamiaceae family. The small molecule components extracted from it have been demonstrated to exhibit a range of beneficial properties, including antiviral, antitumor, antibacterial, antioxidant, and anti-inflammatory activities. Additionally, these components have been shown to protect hepatocytes and nerve cells (Wang et al., 2018). *In vivo* studies have demonstrated that Scutellaria baicalensis Georgi may be capable of treating lipopolysaccharide-induced liver injury in mice by inhibiting the cytokines TNF-α, IL-1β, IL-6, COX-2, iNOS, and NF-κB (Thanh et al., 2015). Lonicerae Japonicae Flos has a long history of use in China as an edible herbal medicine. The extract has been demonstrated to possess anti-inflammatory, bacteriostatic, antiviral, antioxidant, and hepatoprotective effects (Li et al., 2020). The recently identified monoterpenoid components, japopenoid A, japopenoid B, japopenoid C, and a caffeoylquinic acid derivative derived from the extract, have demonstrated anti-hepatoma and anti-HBV activities (Ge et al., 2019). Gardenia jasminoides is another TCM with a range of applications, including the treatment of acute or chronic hepatic diseases, icteric hepatitis, itching skin, eczema, diabetes, and depression (Zhang et al., 2018). Additionally, animal experiments have demonstrated that Gardeniae Fructus can mitigate thioacetamide-induced liver fibrosis in mice *via* the AMPK/SIRT1/NF-κB and Nrf2 signal pathways (Shin et al., 2021). An analysis of the effects of the components of YZH indicates that YZH exerts certain effects on the treatment of jaundice and possesses liver-protective, anti-inflammatory, and antioxidative properties.

Regarding the therapeutic effect of YZH on jaundice, the literature reports that YZH can achieve the effect of relieving jaundice by stimulating the metabolic pathway of bilirubin. The clearance of unconjugated bilirubin and the appearance of conjugated bilirubin in the plasma of rats in the YZH group were faster than those in the control group, and the bilirubin content was also higher than that in the control group (Author Anonymous, 1991). In addition, YZH can be used clinically to treat hemolytic jaundice. Liang et al. (2018) used different doses of YZH to study the effect of YZH on hemolysis in rats with G6-PD activity deficiency. The results showed that YZH did not cause hemolysis in the model rats, and there was no dose dependence. Chen et al. (2015) found in an experiment to explore the effect of YZH oral liquid on the activity of glucose-6-phosphate dehydrogenase (G6-PD) and erythrocyte membrane ATPase that YZH oral liquid can enhance the activity of rat G6-PD enzyme, and the activities of Na^+^-K^+^-ATPase and Ca^2+^-Mg^2+^-ATPase used to maintain the normal morphology of red blood cells also showed an increasing trend. In addition, YZH oral liquid did not cause hemolysis in the model group rats but instead played a certain protective role on the rat erythrocyte membrane.

The overall grade of the evidence is low across the included studies. A total of 23 eligible trials were identified in this review, with a high degree of heterogeneity and publication bias between studies. A subgroup analysis was conducted on the pooled data of total serum bilirubin and jaundice subsiding time, based on different dosages of the intervention herbal medicine. Nevertheless, heterogeneity persisted, with I^2^ values exceeding 50%. In the original studies, the baseline characteristics of the participants and information about the drug manufacturers were seldom provided. However, we did identify disparate sources of YZH injection (produced in different pharmaceutical companies) across the studies, which may account for the existing heterogeneity.

This study evaluated the safety of neonatal jaundice in the YZH injection treatment group from the occurrence of adverse reactions. The reported adverse reactions mainly included fever, diarrhea, vomiting, and rash, which were tolerated by the children or relieved on their own after drug withdrawal. No serious adverse reactions were observed, which did not affect the treatment effect. Because the content of conventional treatment is complex and diverse, the blue light treatment itself will cause adverse reactions such as rash, fever, and diarrhea. YZH itself also has adverse reactions such as diarrhea, vomiting, and rash. Therefore, clinicians are required to pay attention to the adverse reactions when YZH is used in combination or alone and reduce the discomfort or harm caused by the drug to the children. In many cases, the adverse reactions caused by the use of Chinese medicine preparations are related to the patient’s allergic constitution, so it is easy to cause adverse reactions in patients with an allergic constitution. In addition, newborns are more sensitive to drugs, which may be related to a variety of factors, such as lower plasma protein levels in infants and young children than in adults, low plasma protein binding capacity, large body fluid volume, underdeveloped blood-brain barrier, weak liver and kidney function, immature or insufficient enzyme system in newborns, slow drug metabolism, and easy to cause intestinal flora disorders; target organs are more sensitive to certain drugs; therefore, the incidence of adverse reactions is relatively high. YZH preparations are derived from classic Chinese medicine prescriptions and were first marketed in the 1960s and 1970s as injections. Modern pharmacological studies have shown that YZH preparations have the effects of eliminating jaundice and reducing alanine aminotransferase, and are commonly used in the treatment of various types of hepatitis, neonatal jaundice, biliary system diseases, *etc.*, Relevant literature searches have found that there are few reports on YZH preparations based on adverse drug reactions/events. For ADR/ADE caused by YZH preparations, patients generally improve after discontinuation of the drug or symptomatic treatment. Clinically, sufficient attention should be paid and close attention should be paid to the patient’s condition after taking the drug. The researchers (Tai, 2006) found that long-term (12 weeks) administration of YZH injection did not cause chronic toxicity in animals, and the follow-up of jaundiced children in the YZH treatment group for 1 year and 4 months showed no significant difference from the control group in terms of skin and scleral yellowing, serum bilirubin changes, and intellectual development level (Jiang, 2011). Given that a sample size of the study was less than 100 cases, it was not representative, and the intervention measures were complex and diverse (the conventional treatment of the control group included warming, nutritional support, correction of metabolic acidosis, use of blue light irradiation, liver enzyme induction drugs, antibiotics, albumin, microecological preparations, *etc.,*), and the course of treatment and dosage were inconsistent, so the efficacy, long-term safety and dosage of YZH oral solution in the treatment of neonatal jaundice need to be further studied and clarified. Despite synthesizing the data in the meta-analysis, YZH injection has a synergistic effect on phototherapy for babies with neonatal jaundice. It may be a promising adjunct and complementary therapy in clinical practice. Five of the 21 RCTs included in this paper addressed adverse reactions, of which three reported adverse reactions in detail. 182 samples in the treatment group, 16 cases of adverse reactions occurred, including 8 cases of diarrhea, 4 cases of rash, 2 cases of fever, and 2 cases of irritability, the adverse reactions were mild, and all of them improved after symptomatic treatment. In general, different dosages and courses of treatment may produce different efficacy and adverse reactions. The length of treatment in the 21 included papers varied, and the therapeutic dosage of YZH injection in the studies where adverse reactions occurred was ≥2 mL/kg for a 7–10 days course of treatment. In conjunction with the present study, the recommended therapeutic dose for neonates with hyperbilirubinemia is 1–2 mL/kg for 3–7 days.

It is notable that none of the included studies were large-scale, multi-centred trials. The methodological quality of the included trials was found to be inadequate. A comparison of YZH injection with routine treatment demonstrated a superior efficacy of the former in achieving jaundice resolution. The study could broaden its scope to include data from non-Chinese populations to evaluate whether these results apply to different ethnic and cultural groups. Future studies should also compare YZH injection with placebo and with standard therapy to better understand its relative effectiveness. Testing the effect of the herb on newborns with varying levels of serum bilirubin would also provide a clearer indication of its effectiveness in high-risk patients.

## Limitations

The following limitations are inherent to our meta-analysis: (1) The majority of the included articles were retrospective, which increased the risk of information and selection bias; (2) There is publication bias in this study, which may be caused by the following aspects: ① This study extensively searched for literature on YZH injection for the treatment of NH, but there were no negative results published in this study, only positive results were published; ② Some included RCTs, which had relatively small sample sizes and were not representative, and the intervention measures were complex and diverse; ③ In this study, all included literature was Chinese literature, so there may be a certain language publication bias; (3) The clinical heterogeneity of the studies, especially the different sources of YZH injection (produced by other pharmaceutical companies) and the large differences in the therapeutic doses of YZH injection in various literature, may have led to serious heterogeneity; (4) Some included articles did not provide relevant information on gestational age, birth weight, initial bilirubin level, potential causes of hyperbilirubinemia, etc., This may be one of the sources of heterogeneity; (5) There was a lack of evidence for the long-term outcomes and cost-effectiveness of interventions; (6) The evidence for all outcomes was of very low certainty; and (7) This study is not universal. If possible, this herb should be tested on infants of different races. The safety and effectiveness of this therapy need to be confirmed, and the dose range needs to be determined.

## Conclusion

This study highlights the potential benefits of YZH injection in the treatment of NH. Nevertheless, due to the limitations of the quality of evidence, we are unable to make a definitive recommendation regarding the use of YZH injection in conjunction with routine treatment for the management of neonatal jaundice. Although the findings in this study may seem promising for clinical practice, the current evidence is insufficient to recommend YZH injection as a routine treatment for NH. Further research with more robust methodologies and larger, diverse samples is essential to establish YZH injection’s role in the clinical management of neonatal jaundice.

## Data Availability

The original contributions presented in the study are included in the article/Supplementary Material, further inquiries can be directed to the corresponding author.
