# Supplementary material for: Yinzhihuang injection as adjuvant treatment for neonatal hyperbilirubinemia: a systematic review and meta-analysis of randomized clinical trials
Source: Front Pharmacol. 2024 Dec 5;15:1467325. doi: 10.3389/fphar.2024.1467325 (PMC11655195; doi:10.3389/fphar.2024.1467325)
Supplement: Supplementary file 1 [file Table1.doc]

**Appendix**

Specific search strategies

| Database | Time span | Specific search strategy | Number Of hit |
| --- | --- | --- | --- |
| CNKI | inception to April 16, 2024 | (Subject=Neonatal Hyperbilirubinemia OR Subject=neonatal jaundice) AND (Subject=Yinzhihuang Injection) AND (Title/Abstract/Keyword=randomized controlled trial OR Title/Abstract/Keyword=randomized control OR Title/Abstract/Keyword=randomized controlled study OR Title/Abstract/Keyword=randomized OR Title/Abstract/Keyword=RCT) | 54 |
| Wanfang | inception to April 16, 2024 | (Subject=Neonatal Hyperbilirubinemia OR Subject=neonatal jaundice) AND (Subject=Yinzhihuang Injection) AND (Subject=randomized controlled trial OR Subject=randomized control OR Subject=randomized controlled study OR Subject=randomized OR Subject=RCT) | 49 |
| CBM | inception to April 16, 2024 | (("randomized controlled study"[Common Fields:Intelligent] OR "RCT"[Common Fields:Intelligent] OR "randomized control"[Common Fields:Intelligent] OR "randomized"[Common Fields:Intelligent]) OR ("randomized controlled trial"[Unweighted:Extended])) AND ("Yinzhihuang Injection"[Unweighted:Extended]) AND (("neonatal jaundice"[Common Fields:Intelligent]) OR ("Hyperbilirubinemia, neonate"[Unweighted:Extended])) | 34 |
| VIP | inception to April 16, 2024 | (((Title/Keyword=Neonatal Hyperbilirubinemia OR Title/Keyword=neonatal jaundice) AND Title/Keyword=Yinzhihuang Injection) AND ((((Abstract=randomized control OR Abstract=randomized controlled trial ) OR Abstract=randomized controlled study ) OR Abstract=RCT) OR Abstract=randomized)) | 46 |
